# A Time-Calibrated Mitogenome Phylogeny of Catfish (Teleostei: Siluriformes)

**DOI:** 10.1371/journal.pone.0166988

**Published:** 2016-12-01

**Authors:** Ilias Kappas, Spiros Vittas, Chrysoula N. Pantzartzi, Elena Drosopoulou, Zacharias G. Scouras

**Affiliations:** Department of Genetics, Development & Molecular Biology, School of Biology, Aristotle University of Thessaloniki, Thessaloniki, Greece; National and Kapodistrian University of Athens, GREECE

## Abstract

A very significant part of the world’s freshwater ichthyofauna is represented by ancient, exceptionally diverse and cosmopolitan ray-finned teleosts of the order Siluriformes. Over the years, catfish have been established as an exemplary model for probing historical biogeography at various scales. Yet, several tantalizing gaps still exist in their phylogenetic history, timeline and mode of diversification. Here, we re-examine the phylogeny of catfish by assembling and analyzing almost all publicly available mitogenome data. We constructed an ingroup matrix of 62 full-length mitogenome sequences from 20 catfish families together with four cypriniform outgroups, spanning 15,557 positions in total. Partitioned maximum likelihood analyses and Bayesian relaxed clock dating using fossil age constraints provide some useful and novel insights into the evolutionary history of this group. Loricarioidei are recovered as the first siluriform group to diversify, rendering Neotropics the cradle of the order. The next deepest clade is the South American Diplomystoidei placed as a sister group to all the remaining Siluroidei. The two multifamilial clades of “Big Asia” and “Big Africa” are also recovered, albeit nodal support for the latter is poor. Within “Big Asia”, Bagridae are clearly polyphyletic. Other interfamilial relationships, including Clariidae + Heteropneustidae, Doradidae + Auchenipteridae and Ictaluridae + Cranoglanididae are robustly resolved. Our chronogram shows that siluriforms have a Pangaean origin, at least as far back as the Early Cretaceous. The inferred timeline of the basal splits corroborates the “Out-of-South America” hypothesis and accords well with the fossil record. The divergence of Siluroidei most likely postdated the final separation of Africa and South America. An appealing case of phylogenetic affinity elaborated by biogeographic dispersal is exemplified by the Early Paleogene split between the Southeast Asian Cranoglanididae and Ictaluridae, with the latter radiating into North America’s freshwater realm by Eocene. The end of Cretaceous probably concludes the major bout of diversification at the family level while with the dawn of the Cenozoic a prolific radiation is evident at the generic level.

## Introduction

An impressive proportion of more than 40% of currently recognized fish species are confined to a trivial 0.8% of the earth’s surface, namely freshwater ecosystems [1]. About two-thirds of fish living in freshwater belong to a single clade, series Otophysi of the superorder Ostariophysi. Otophysan fish are found abundantly in all continents except Antarctica and are divided into four orders: Characiformes (piranhas, tetras), Cypriniformes (minnows, carps, barbs, suckers, loaches), Gymnotiformes (electric eels and relatives), and Siluriformes (catfishes) [2].

The order Siluriformes constitutes an exceptionally diverse and speciose natural group of primarily freshwater ray-finned fish. Currently, 36 families and over 3,000 species are recognized [3] rendering catfishes among the most diverse vertebrate orders (approximately 1 in 10 actinopterygians or 1 in 20 vertebrates is a catfish). With an extraordinary diversity in size, a unique repertoire of behavioral, physiological and ecological traits [4] and significant economic value in subsistence fisheries, pet trade and angling [5], catfishes have been the focus of varied research for many years. From an evolutionary perspective in particular, the order Siluriformes is considered as a model system for the investigation of historical continental relationships and biogeographical patterns (e.g. vicariance vs dispersal). Conducive to such research using modern phylogenetic methods and divergence dating techniques are a plethora of characteristics including an ancient origin of the order, a global yet fragmented distribution of several families, secondary transitions to marine waters (Ariidae, Plotosidae and, to a lesser extent, Aspredinidae), alleged limited trans-marine dispersals as well as a fairly rich fossil record even from Eocene deposits of Antarctica [6–12]. It is thus obvious that studies on multi-scale lineage radiation and diversification in otophysan fish or their subdivisions appeal to a wide audience and have been rather influential in resolving intriguing questions in major parts of the vertebrate tree [13–14].

Morphological [15–16] as well as molecular data [2, 17] clearly support the monophyly of Siluriformes. The earliest fossils assigned to extant siluroid families date from the Maastrichtian and Campanian (ca. 68–73 Ma) of Argentina, Bolivia and Brazil (see [7, 18]), although molecular clocks imply a much older origin (97–180 Ma) of the group [9, 11–12, 19]. Despite the diversity of catfishes, interfamilial relationships still remain controversial and not fully resolved. Diogo & Peng [20] presented a comprehensive review of higher-level siluriform relationships. Excluding cladistic analyses based on morphological characters, current knowledge regarding the higher-level phylogeny within siluriforms stems mainly from two molecular studies that have specifically focused on catfish: Hardman [21] analyzed cytochrome *b* sequences from 170 species of catfish from 29 of 33 families while Sullivan et al. [17] used *rag*1 and *rag*2 nuclear gene sequences from 110 catfish species representing 36 of 37 families. In a third study, Betancur-R et al. [22] used 21 molecular markers to produce a comprehensive molecular phylogeny of all major lineages of bony fishes, including 32 siluriform families. Thus, considerable agreement exists i) on the close relationship between North American ictalurids and Southeast Asian *Cranoglanis*, ii) on the basal position within the order of the South American Loricarioidei and Diplomystidae, iii) on a number of multifamilial clades [e.g. (Clariidae, Heteropneustidae); (Pimelodidae, Pseudopimelodidae, Heptapteridae); (Doradidae, Auchenipteridae)], iv) on a “Big Asia” clade (Bagridae, Horabagridae, Akysidae, Amblycipitidae, Sisoridae, Erethistidae), and v) on a “Big Africa” clade (Mochokidae, Malapteruridae, Amphiliidae, Claroteidae, Schilbidae). Nevertheless, the phylogenetic relationships of a number of families such as Siluridae, Schilbeidae, Malapteruridae, Bagridae, Mochokidae and Plotosidae have so far consistently resisted resolution.

The diversification of Siluriformes is also unresolved and several biogeographic scenarios exist describing the sequence of events that shaped the modern-day distribution of the different families. As a consequence, both the timeline and mode (continental drift *vs* intracontinental dispersal) of siluriform radiation are as yet uncertain [8, 11–12, 19, 21]. In an effort to elucidate the puzzling geographical distribution of catfishes, Diogo [8] evaluated the two main biogeographic hypotheses describing their diversification and also proposed a third one based on his own analysis of 440 morphological characters from 32 extant catfish families. In the “traditional hypothesis”, siluriforms have a Gondwanan origin while their extended distribution is the result of dispersal through available Paleocene and Eocene connections and continental passages to Laurasian areas. The “marine hypothesis” essentially postulates a marine origin and dispersion leading to the current worldwide distribution of catfishes. Diogo’s [8] own proposal is in fact an amalgam of the existing hypotheses in a complex biogeographic arrangement involving pre- and post-drift dispersals, vicariant events and possible marine migrations. Most importantly, however, it identifies South America as the cradle of the whole clade and extends its origin to the Early Cretaceous.

With these in mind we set out to re-examine the phylogenetic relationships and timing of diversification of siluriforms. We explicitly focused on the phylogenetic signal harbored in the mitochondrial genome. We used here, for the first time, all available mitogenome sequences of Siluriformes in order to build a comprehensive maximum likelihood phylogeny and estimate divergence times from fossil-based calibrations in relaxed-molecular clock analyses.

## Materials and Methods

### Dataset assembly and alignment

We downloaded all available siluriform mitogenomes using the METAMiGA database (METAzoan Mitochondrial Genomes Accessible database, http://amiga.cbmeg.unicamp.br/). In total, 62 full-length-only RefSeq mtDNA sequences were retrieved corresponding to 20 catfish families (Table 1). Listings were cross-checked with the Organelle Genome Resources of the National Center for Biotechnology Information (NCBI, http://www.ncbi.nlm.nih.gov) as well as the Mitochondrial Genome Database of Fish (MitoFish, http://mitofish.aori.u-tokyo.ac.jp) [49]. In addition, all species names were checked against the Catalog of Fishes [50]. Four cypriniform sequences were also retrieved to be used as outgroups. All full-length sequences were trimmed to 12 protein-coding genes, the two rRNA genes (12S and 16S rRNA), and the 22 tRNAs. We excluded the D-loop region in order to avoid saturation problems due to fast evolutionary rates as well as the ND6 gene, given evidence for heterogeneous base composition and poor phylogenetic signal [51]. During data analysis and manuscript preparation several additional catfish mitogenomes became available. Two of these new mitogenomes belong to families (Plotosidae and Pseudopimelodidae) unsampled in our current dataset. Previous analyses (e.g. [17]) have recovered these families deep within other clades for which we have representative sequences and their absence from our dataset is therefore not expected to have an effect on our hypothesis of overall siluriform phylogeny.

**Table 1:** List of siluriform and outgroup species used in this study.

Family	Species	GenBank accession No.	Reference
Amblycipitidae	*Liobagrus anguillicauda*	NC_021602	unpublished
	*Liobagrus kingi*	NC_020337	[23]
	*Liobagrus marginatoides*	NC_021122	[24]
	*Liobagrus marginatus*	NC_022923	[25]
	*Liobagrus nigricauda*	NC_021407	[26]
	*Liobagrus obesus*	NC_008232	[27]
Amphiliidae	*Amphilius* sp.	NC_015746	[11]
Aspredinidae	*Bunocephalus coracoideus*	NC_015811	[11]
Auchenipteridae	*Centromochlus perugiae*	NC_015748	[11]
	*Tetranematichthys quadrifilis*	NC_015743	[11]
Bagridae	*Auchenoglanis occidentalis*	NC_015809	[11]
	*Hemibagrus macropterus*	NC_019592	[28]
	*Hemibagrus spilopterus*	NC_023222	unpublished
	*Leiocassis crassilabris*	NC_021394	[29]
	*Leiocassis longirostris*	NC_014586	[30]
	*Mystus rhegma*	NC_023223	unpublished
	*Pelteobagrus eupogon*	NC_018768	[31]
	*Pelteobagrus fulvidraco*	NC_015888	[32]
	*Pelteobagrus nitidus*	NC_014859	[33]
	*Pelteobagrus vachellii*	NC_014862	unpublished
	*Pseudobagrus albomarginatus*	NC_022726	[34]
	*Pseudobagrus brevicaudatus*	NC_021393	[35]
	*Pseudobagrus brevicorpus*	NC_015625	[36]
	*Pseudobagrus ondon*	NC_022725	[37]
	*Pseudobagrus tokiensis*	NC_004697	[2]
	*Pseudobagrus trilineatus*	NC_022705	unpublished
	*Pseudobagrus truncatus*	NC_021395	[38]
	*Pseudobagrus ussuriensis*	NC_020344	[39]
	*Rita rita*	NC_023376	unpublished
Callichthyidae	*Corydoras rabauti*	NC_004698	[2]
Clariidae	*Clarias* sp.	NC_015749	[11]
Cranoglanididae	*Cranoglanis bouderius*	NC_008280	[40]
Diplomystidae	*Diplomystes nahuelbutaensis*	NC_015823	[11]
Doradidae	*Amblydoras gonzalezi*	NC_015745	[11]
Heteropneustidae	*Heteropneustes fossilis*	NC_015827	[11]
Ictaluridae	*Ictalurus punctatus*	NC_003489	[41]
Loricariidae	*Pterygoplichthys disjunctivus*	NC_015747	[11]
Malapteruridae	*Malapterurus electricus*	NC_015833	[11]
Mochokidae	*Synodontis schoutedeni*	NC_015808	[11]
Pangasiidae	*Pangasianodon gigas*	NC_006381	[42]
	*Pangasianodon hypophthalmus*	NC_021752	[43]
	*Pangasius larnaudii*	NC_015839	[11]
Pimelodidae	*Pimelodus pictus*	NC_015797	[11]
Schilbeidae	*Pareutropius debauwi*	NC_015837	[11]
Siluridae	*Silurus asotus*	NC_015806	[11]
	*Silurus glanis*	NC_014261	[44]
	*Silurus lanzhouensis*	NC_015650	unpublished
	*Silurus meridionalis*	NC_014866	unpublished
	*Silurus soldatovi*	NC_022723	unpublished
Sisoridae	*Bagarius yarrelli*	NC_021606	unpublished
	*Creteuchiloglanis kamengensis*	NC_021599	unpublished
	*Euchiloglanis kishinouyei*	NC_021598	unpublished
	*Exostoma labiatum*	NC_021601	unpublished
	*Gagata dolichonema*	NC_021596	unpublished
	*Glaridoglanis andersonii*	NC_021600	unpublished
	*Glyptosternon maculatum*	NC_021597	unpublished
	*Glyptothorax fokiensis fokiensis*	NC_018769	[45]
	*Glyptothorax trilineatus*	NC_021608	unpublished
	*Oreoglanis macropterus*	NC_021607	unpublished
	*Pareuchiloglanis gracilicaudata*	NC_021603	unpublished
	*Pseudecheneis sulcata*	NC_021605	unpublished
	*Pseudexostoma yunnanensis*	NC_021604	unpublished
**Outgroup (Cypriniformes)**			
Catostomidae	*Catostomus commersonii*	NC_008647	[46]
Cobitidae	*Cobitis striata*	NC_004695	[2]
Cyprinidae	*Cyprinus carpio*	NC_001606	[47]
	*Danio rerio*	NC_002333	[48]

The alignment of the concatenated dataset was generated through the METAMiGA server using ClustalW [52]. As a quality control, we inspected substitution saturation for each partition (see below) in DAMBE through Xia et al.’s test [53] and constructed saturation plots of the observed number of transitions and transversions against corrected genetic distances. Saturation is inferred when the index of substitution saturation (*I*_*SS*_) is either larger or not significantly smaller than the critical value (*I*_*SS*.*C*_). The final alignment consisted of 10,994 positions for the 12 protein-coding genes, 2,846 positions for the rRNA genes, and 1,717 positions for the 22 tRNAs (total 15,557 positions). To avoid overparameterization and poor mixing in subsequent Bayesian analyses we partitioned the concatenated alignment into four partitions, namely first and second codon positions, third codon positions, rRNAs, and tRNAs (designated as 12–3–R—T), following the recommendations of Li et al. [54] and Inoue et al. [55] about functional constraints on sequence evolution for codon positions. Nonetheless, we also assessed clade stability by generating topologies based on alternative combinations of partitions.

### Maximum likelihood phylogenetic analyses

Maximum likelihood (ML) analysis of the partitioned dataset was implemented with the graphical interface raxmlGUI 0.93 [56] of RAxML-VI-HPC [57]. We used jModelTest ver. 2.1.4 [58] and the Akaike Information Criterion (AIC) to choose the best model of nucleotide substitution for each partition. RAxML was set up with the -f a option that executes a rapid bootstrap (BS) analysis using the selected model (GTR + Γ + I, four discrete rate categories) with 1,000 replications. In particular, the algorithm first performs BS analysis using GTRCAT (a GTR approximation with optimization of individual per-site substitution rates and classification of those individual rates into the number of rate categories) and then uses every fifth BS tree as a starting point for another ML search using the GTR + Γ + I model, saving the top 10 best-scoring ML trees. It follows calculation of likelihood scores (slow ML search) for those 10 trees and assignment of BS probabilities on the best-scoring ML tree. Branch lengths were calculated and optimized independently for each partition.

### Divergence time estimation

We used BEAST v.1.8.0 [59] to infer divergence times and their 95% credibility intervals under a Bayesian relaxed-clock framework incorporating an uncorrelated log-normal rate variation model. The input file was constructed in BEAUTi. For each partition, the GTR + Γ + I model of sequence evolution was used. All other parameters were linked (clock and tree models) with rates of cladogenesis modeled through the Yule process. Two independent, identical analyses of 8 × 10^7^ generations were run, each initiated with a random starting tree. Trees and time estimates were sampled every 1,000 generations. Chain convergence was assessed in Tracer v1.6 through the effective sample sizes (ESS) values (>200) of each parameter. We used TreeAnnotator v1.8.0 to discard 20% of samples as burn-in and summarize the information of the remaining samples of trees onto a maximum clade credibility chronogram with posterior probabilities of nodes, mean divergence times and 95% age credibility intervals.

### Fossil-based calibrations

To calibrate our chronogram we selected six fossils from the teleost fossil record. We assigned exponential prior age distributions on selected nodes based on the assumption that fossils provide reliable group ages to a reasonable degree. We specified hard lower bounds, representing the respective fossil age, and means in such a way that 95% of the probability was contained between the hard lower bounds and a soft upper bound defined by the age of the crown-group Ostariophysi (see below). The following fossil calibrations were used:

1. tMRCA of root: The oldest fossil of the crown-group Ostariophysi is the stem gonorynchiform †*Rubiesichthys gregalis* [60–61] from the Berriasian of Spain (145.5–140.2 Ma). Lower age constraint of root = 140.2 Ma. Soft upper bound = 145.5 Ma (exponential distribution mean = 1.45).
2. tMRCA of the crown-group Siluroidei: Siluroid fossils from the Campanian stage (70.6–83.5 Ma) in South America [18]. Lower age constraint of the crown-group Siluroidei = 70.6 Ma. Soft upper bound = 140.2 Ma (exponential distribution mean = 18.86).
3. tMRCA of Cobitoidei: The earliest cobitoid fossils from the family Catostomidae date back to the Early Paleocene (61.1–65.5 Ma) [62–64]. Lower age constraint of Cobitoidei = 61.1 Ma. Soft upper bound = 140.2 Ma (exponential distribution mean = 21.45).
4. tMRCA of the clade (*Ictalurus*, *Cranoglanis*): The oldest ictalurid stem fossil *Astephus* sp. [65] from the Polecat Bench Formation, Wyoming dates back to the Early Paleocene (63–65 Ma). Lower age constraint of clade (*Ictalurus*, *Cranoglanis*) = 63.0 Ma. Soft upper bound = 140.2 Ma (exponential distribution mean = 20.94).
5. tMRCA of the clade (*Danio*, *Cyprinus*): The †*Parabarbus* fossil (Cyprinidae) from the Ypresian of the Obailinskaya Formation, Zaissan Basin, Kazakhstan (51–49 Ma) is the oldest crown cyprinid [66]. Lower age constraint of clade (*Danio*, *Cyprinus*) = 49 Ma. Soft upper bound = 140.2 Ma (exponential distribution mean = 24.73).
6. tMRCA of the clade (*Clarias*, *Heteropneustes*): First appearance of the African Clariidae in the Lower Eocene (34–56 Ma) [7]. Lower age constraint of clade (*Clarias*, *Heteropneustes*) = 34 Ma. Soft upper bound = 140.2 Ma (exponential distribution mean = 28.8).

## Results

### Sequence statistics and substitution saturation

Of the 15,557 positions of the concatenated alignment, 8,755 and 7,358 were variable and parsimony-informative, respectively. Results of substitution saturation tests [53] for each of the four partitions (12–3–R—T) did not reveal any significant sequence saturation with the *I*_*SS*_ indices being significantly lower than the critical *I*_*SS*.*C*_ values in all cases. The same was true for the concatenated alignment considered as a whole. However, in saturation plots for third codon positions, a divergence plateau could be seen at corrected genetic distance values above 0.30 substitutions/site (S1 Fig).

### Phylogenetic relationships

**Fig 1:**
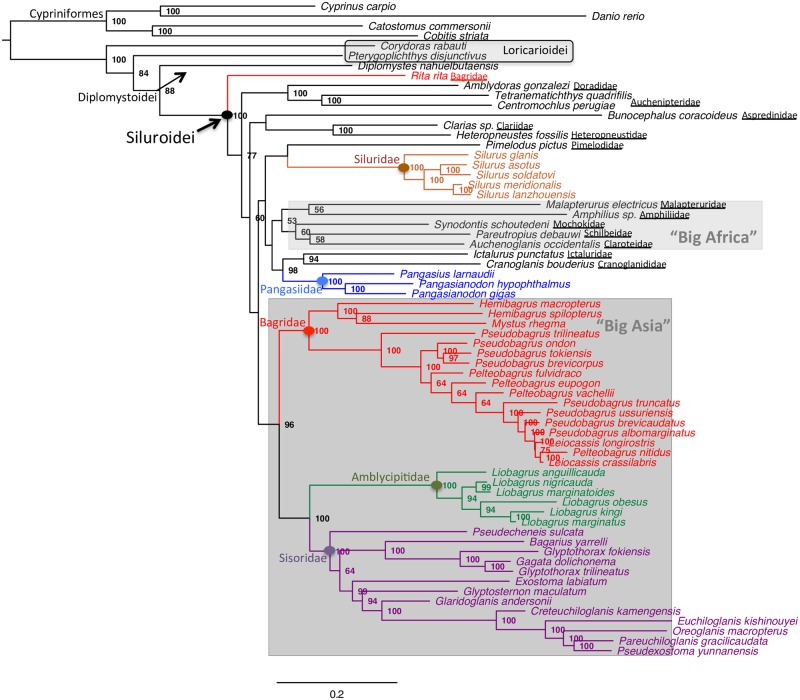
Best-scoring maximum-likelihood tree of 62 ingroup siluriforms and four cypriniform outgroup species obtained from the partitioned (12–3–R—T) RAxML analysis of 15,557 positions. Branch lengths are proportional to the number of inferred substitutions. Numbers at nodes are bootstrap proportions (if ≥50%) based on 1,000 replicates. Monophyletic families and the polyphyletic Bagridae are colored. The lineage leading to *Rita rita* is also colored to indicate polyphyly of Bagridae (see text for details). Loricarioidei and the two multifamilial clades, “Big Africa” and “Big Asia”, are demarcated by grey boxes.

The best-scoring maximum likelihood phylogeny of Siluriformes based on the 12–3–R—T partitioning (first and second codon positions, third codon positions, rRNAs, tRNAs) is shown in Fig 1. A number of important features are revealed. The Neotropical Loricarioidei are recovered as the first siluriforms to diversify and especially *Corydoras rabauti* of the family Callichthyidae. The next deepest clade is the South American Diplomystoidei (*Diplomystes nahuelbutaensis*), which is placed as a sister group to all the remaining Siluroidei. Within Siluroidei, the “Big Asia” clade composed of Bagridae, Amblycipitidae and Sisoridae is strongly supported (96% bootstrap). Bagridae are rendered polyphyletic as gauged by the topological placement of *Rita rita* (Fig 1). The second multifamilial clade, “Big Africa”, is also recovered although with poor nodal support. It includes the families Malapteruridae (*Malapterurus electricus*), Amphiliidae (*Amphilius* sp.), Mochokidae (*Synodontis schoutedeni*), Schilbeidae (*Pareutropius debauwi*) and Claroteidae (*Auchenoglanis occidentalis*).

Families with increased sampling like Siluridae and Pangasiidae were recovered as monophyletic while other traditionally recognized clades were also robustly resolved: Clariidae + Heteropneustidae (*Clarias* sp. + *Heteropneustes fossilis*), the South American Doradidae + Auchenipteridae (*Amblydoras gonzalezi* + *Tetranematichthys quadrifilis*, *Centromochlus perugiae*), and Ictaluridae + Cranoglanididae (*Ictalurus punctatus* + *Cranoglanis bouderius*). The latter clade, along with the family Pangasiidae, was recovered as the sister-group of the “Big Africa” clade, albeit with weak nodal support.

Alternative topologies were reconstructed based on different combinations of partitions, namely 12+3 (two partitions; first and second codon positions, third codon positions), 12 (a single partition; first and second codon positions only), 1–2–3 (three partitions; first, second, and third codon positions) (S2–S4 Figs). In all cases, the main clades described earlier were consistently robust and stable and only subtle differences occurred (with reference to the 12–3–R—T partition) concerning interfamilial relationships at the already weakest parts of the best-scoring phylogeny. The most salient feature in those parts of the alternative topologies was the significant drop in node support, the grouping of *Pimelodus pictus* (Pimelodidae) with the Clariidae + Heteropneustidae clade, the grouping of the South American *Bunocephalus coracoideus* (Aspredinidae) with the other South American clade Doradidae + Auchenipteridae, and the breakup of the “Big Africa” clade following complete removal of third codon positions (partition 12; S3 Fig).

### Divergence time estimates

We have estimated divergence times and 95% age credibility intervals by assigning exponential prior age distributions on selected nodes, calibrated using fossil evidence.

**Fig 2:**
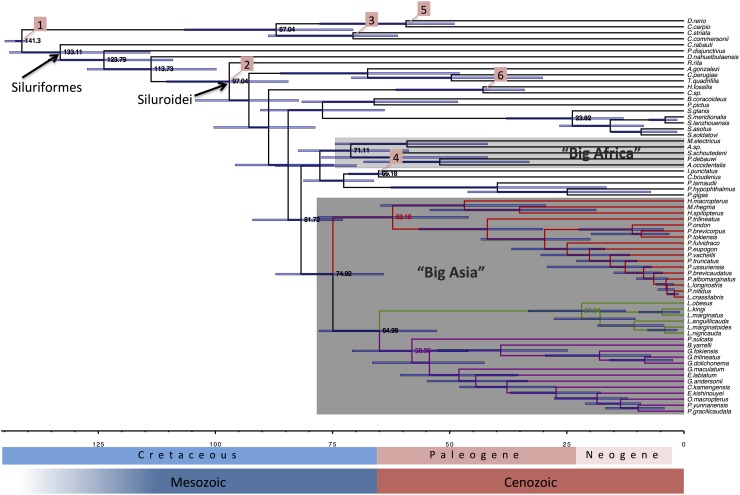
Chronogram derived from the Bayesian relaxed-clock analysis using the 12–3–R—T partitioned dataset and calibrated with six fossil-based constraints following exponential distributions (see text for details). Horizontal timescale is in million years before present (Ma). Blue horizontal bars at nodes are 95% age credibility intervals. Boxed numbers 1–6 indicate the respective nodes calibrated using fossil constraints. Numbers at selected nodes indicate node ages in Ma.

The inferred ages of diversification within Siluriformes are shown in Fig 2. The origin of Siluriformes is placed at 133.1 Ma whereas the most basal splits, the divergence of Loricariidae (*Pterygoplichthys disjunctivus*, 123.8 Ma) and Diplomystidae (*Diplomystes nahuelbutaensis*, 113.7), postdate the final stages of separation of Laurasia and Gondwana (about 140 Ma). The divergence of the crown group Siluroidei (97 Ma) roughly coincides with the complete separation of Africa and South America (about 100 Ma). Below this level, most families had already diversified by the end of Cretaceous (66 Ma). With the dawn of the Cenozoic, a prolific radiation is evident at the generic level (see Fig 2). The two most prominent multifamilial clades, “Big Africa” and “Big Asia” diverged almost concurrently, at 71.1 and 74.9 Ma, respectively. Within the “Big Asia” clade the diversification of the major families Bagridae and Sisoridae occurred just after the K-Pg boundary (<66 Ma). Shark catfish of the family Pangasiidae, torrent catfish (Amblycipitidae) and the Eurasian silurids (the nominate family of the order) diverged at a later time (39.8, 21 and 23.8 Ma, respectively). The intercontinental catfish clade composed of the North American ictalurids (*Ictalurus punctatus*) and the East Asian *Cranoglanis bouderius* diverged 65.1 Ma, with the outset of Paleogene. Roughly (i.e. excluding the Asian bagrid *Rita rita* and the Asian-African clade of *Clarias* sp. + *Heteropneustes fossilis*), the dominant feature of the chronogram in Fig 2 is the initial split of South American lineages followed by diversification of African and Asian clades within a short window of time past the 85 Ma marker.

## Discussion

### Interfamilial relationships

The current work is, to our knowledge, the first attempt to explicitly address the phylogeny and timing of diversification of the order Siluriformes based on an all-inclusive mitogenomic dataset. Previous studies [17, 21] have assessed the historical relationships of catfish using less sequence data (one or two mitochondrial/nuclear loci) without providing node age estimates. A notable difference, although not focusing exclusively on Siluriformes, is the work of Betancur-R et al. [22] who used 21 different molecular markers (one mitochondrial and 20 nuclear) and provided a time-calibrated phylogeny of bony fishes.

The critical questions in siluriform phylogeny have traditionally centered on the basal divergences, the recovered nodal support of the different clades and the constituent genera of multifamilial clades. In accordance with the results of recent studies [11–12, 17], monophyly of the suborder Siluroidei is strongly supported (Fig 1) as well as a basal placement of the suborder Loricarioidei. Thus, the mitogenomic historical signal fully supports the Neotropics as the center of origin of the order, a finding also upheld by the fact that catfish exhibit their highest diversity in South and lower Central America [17]. The ancient divergence of Loricarioidei is followed by the split of another clade, the southern South American endemic diplomystids, branching off as sister to Siluroidei and bearing the most pleisiomorphic characters of all siluriforms. Jointly, the above basal splits corroborate the “Out-of-South America” hypothesis proposed by Sullivan et al. [17] and firmly identify the center of deep diversification of catfish. Although the extinct Hypsidoridae from the Eocene of North America are still a problematic ancient clade with inconstant topological placement (more basal or more derived; see [20]), from a molecular perspective the issue of the basal branching pattern and order in Siluriformes is most likely settled. With reference to that, a problematic feature in our analysis appears to be the paraphyletic arrangement of the two loricarioid representatives (*Corydoras rabauti*, Callichthyidae and *Pterygoplichthys disjunctivus*, Loricariidae) which has not been reported previously. This result is most probably due to the comparatively poor taxon sampling of this suborder and does not represent a credible topology, although it may have implications for the chronology of the basal siluriform nodes (see below). For example, in the densest marker-wise phylogeny so far, that of Betancur-R et al. [22], Loricarioidei form a clade in which Callichthyidae and Loricariidae are recovered as sister-groups and Nematogenyidae and Trichomycteridae (unsampled here) are the first lineages to diverge (in that order).

Regarding the topological support of different groupings and the interclade relationships within the order, there appears to be extensive concordance between our results and those of previous studies (for a review see [20]). In particular, the clade including the genus *Heteropneustes* and clariids (*Clarias* sp.) is firmly established and it is also backed by both morphological [67] and molecular synapomorphies [17]. This tight relationship is solid throughout our analyses although it differs in its frail grouping with *Pimelodus pictus* in the topologies reconstructed by alternative partitions (S2–S4 Figs).

Another clade enjoying high support and showing consistency in all analyses (Fig 1 and S2–S4 Figs) is that composed of the South American Doradidae (*Amblydoras gonzalezi*) and Auchenipteridae (*Tetranematichthys quadrifilis* + *Centromochlus perugiae*). This affinity has been a recurrent feature in morphological [20] as well as molecular examinations [17, 21]. For the latter, it is interesting to note that Doradidae + Auchenipteridae appeared closely related to the South American Aspredinidae as it is also seen here by the firm joining of the aspredinid *Bunocephalus coracoideus* to the above clade in the alternative partition analyses (S2–S4 Figs).

A number of families are recovered as clearly monophyletic: a) the Asian Sisoridae and Amblycipitidae, which are arranged as sister groups, b) the South-Southeast Asian Pangasiidae which share a close relationship with the monotypic Southeast Asian family Cranoglanididae (*Cranoglanis bouderius*) and the North American endemic Ictaluridae (*Ictalurus punctatus*), and c) the Eurasian Siluridae with unresolved connections to other catfish families. A puzzling relationship that has been volleyed back and forth from the early years until quite recently is that between ictalurids and cranoglanidids. Compelling evidence was finally provided by the morphological study of Diogo [8] and later corroborated by the molecular investigations of Hardman [21] and Sullivan et al. [17]. In our analyses, this clade is consistently recovered, being immune to data partitioning (Fig 1 and S2–S4 Figs). It is fair to say that the grouping of ictalurids and cranoglanidids epitomizes the merits of the crown group Siluriformes as a model system for the study of the basic drivers (vicariance and/or dispersal) of intercontinental diversification. Notably, there exists another strong case of “Intercontinentals” (see also [17]), that of the African-Asian ichthyofaunal pair of *Clarias* sp. + *Heteropneustes fossilis* whereas, in an interesting twist and unlike Characiformes (see [68]), trans-Atlantic African-South American pairs are not observed.

The presence of the two large multifamilial clades, “Big Africa” and “Big Asia”, is the preponderant feature of catfish phylogeny seen as a whole. As properly put by Sullivan et al. [17], the prolific diversification within these clades accounts for the overriding majority (projected to reach >75% on average when unresolved families are phylogenetically placed) of catfish genera and species in their respective continents. This highlights the importance of intracontinental radiations in producing the overwhelming diversity of catfish recorded at the present time.

### Catfish diversification

In a temporal context and with reference to other molecular estimates as well as the fossil record, a series of landmarks can be outlined in the historical biogeography of siluriforms.

First, recent studies [8, 11, 12, 14, 19, 63], as well as the present work, agree that siluriforms (and in fact all modern otophysan lineages) have a Pangaean origin, at least as far back as the Early Cretaceous. Our molecular estimate of 133.1 Ma (95% HPD 113.95–143.98; Fig 2) for that node (origin of Siluriformes) is the least discrepant from the timespan implied by fossil evidence of any mitogenome study to date. According to the fossil record, the earliest catfish fossil marking the lower boundary for the age of the crown group Siluriformes dates back to 83.5–88.6 Ma [69]. On the upper end, the oldest fossil of the crown-group Ostariophysi is the stem gonorynchiform †*Rubiesichthys gregalis* (140.2–145.5 Ma) [60–61]. In that sense, the inferred siluriform age reconciles molecular estimates and fossil dates to a great extent and avoids the need to invoke “ghost” lineages in order to interpret apparent gaps in the fossil record. This is also surprising given the fact that mitochondrial data tend to provide overestimates of divergence times, especially at deeper nodes due to the effects of substitution saturation (see [70]). In addition, Betancur-R et al. [22], with a richer sampling of loricarioids than ours, provided an age estimate for the divergence of Siluriformes close to 117 Ma. Taking all the above evidence together, we may safely conclude that siluriforms diverged prior to the separation of Africa from South America and not later than our inferred estimate (ca. 135 Ma).

Second, the divergence of Siluroidei (97 Ma, 95% HPD 84.45–110.43; Fig 2) most likely postdated the final separation of Africa and South America. The window of time defined by this node and down to the next one, separating the South American clade of Doradidae (*Amblydoras gonzalezi*) and Auchenipteridae (*Tetranematichthys quadrifilis* + *Centromochlus perugiae*) (92.86 Ma, 95% HPD 82.22–104.29), is slightly problematic as the credibility intervals overlap with the severance of Gondwana’s two major subcontinents (Africa and South America). It is possible enough that the node ages derived here have been affected by the rogue behavior (e.g. long-branch attraction) of *Rita rita*, *Bunocephalus coracoideus* and *Pimelodus pictus* as discussed earlier.

Third, the divergence between the North American Ictaluridae (*Ictalurus punctatus*) and the Southeast Asian Cranoglanididae (*Cranoglanis bouderius*) at 65.18 Ma (95% HPD 63–71.58; Fig 2) provides a credible scenario for the origin of North America’s present-day catfish diversity. This intercontinental clade is a fine example of phylogenetic affinity elaborated by biogeographic dispersal. Favorable evidence is consistent and multiple: a) geological evidence on the North America’s Early Paleogene (ca. 60 Ma) connection to Northeastern Asia via the Bering Strait [71], b) palaeontologic evidence on the oldest ictalurid stem fossil *Astephus* sp. [65] from Wyoming at the same epoch, and c) additional examples of North American-East Asian freshwater fish connections like the polyodontid paddlefish [72] and the catostomid suckers [73].

Finally, the end of Cretaceous probably concludes the major bout of diversification at the family level. Prior to that, the split between the two multifamilial clades, “Big Asia” and “Big Africa”, at 81.72 Ma (95% HPD 72.87–92.08) is likely accounted for by dispersal from Africa and through shallow epicontinental Tethyan sea corridors to Asia. However, inferences about this node should be based on additional data (taxon and/or character sampling) leaning towards the “Big Africa” clade and they are also further complicated by the closer relationship of the South-Southeast Asian Pangasiidae (*Pangasius larnaudii*, *Pangasianodon hypophthalmus* and *Pangasianodon gigas*) with “Big Africa” than with other “Big Asia” representatives.

## Conclusions

Siluriforms comprise a basic component of the world’s freshwater realm. They evolved in Pangaean Neotropical settings in the Early Cretaceous, diverged basally during the African-South American severance and diversified mostly intracontinentally until the K-Pg boundary. Cladogenetic events during the Cenozoic account for the bulk of today’s generic diversity in the resulting landmasses. This crown group, along with other otophysan relatives, still poses inviting questions to the wide audience of historical biogeographers seeking answers in the vertebrate tree of life.

## Supporting Information

S1 Fig Substitution saturation plot for third codon positions of the 12 protein-coding genes. Transitions (s, in blue) and transversions (v, in green) are plotted against GTR distances.
(TIFF)

S2 Fig Best-scoring maximum-likelihood tree of 62 ingroup siluriforms and four cypriniform outgroup species obtained from the 12+3 partition. Branch lengths are proportional to the number of inferred substitutions. Numbers at nodes are bootstrap proportions based on 1,000 replicates.
(TIFF)

S3 Fig Best-scoring maximum-likelihood tree of 62 ingroup siluriforms and four cypriniform outgroup species obtained from the 12 partition. Branch lengths are proportional to the number of inferred substitutions. Numbers at nodes are bootstrap proportions based on 1,000 replicates.
(TIFF)

S4 Fig Best-scoring maximum-likelihood tree of 62 ingroup siluriforms and four cypriniform outgroup species obtained from the 1–2–3 partition. Branch lengths are proportional to the number of inferred substitutions. Numbers at nodes are bootstrap proportions based on 1,000 replicates.
(TIFF)
