# Supplementary figures and images for: A Time-Calibrated Mitogenome Phylogeny of Catfish (Teleostei: Siluriformes)
Source: PLoS One. 2016 Dec 1;11(12):e0166988. doi: 10.1371/journal.pone.0166988 (PMC5132296; doi:10.1371/journal.pone.0166988)

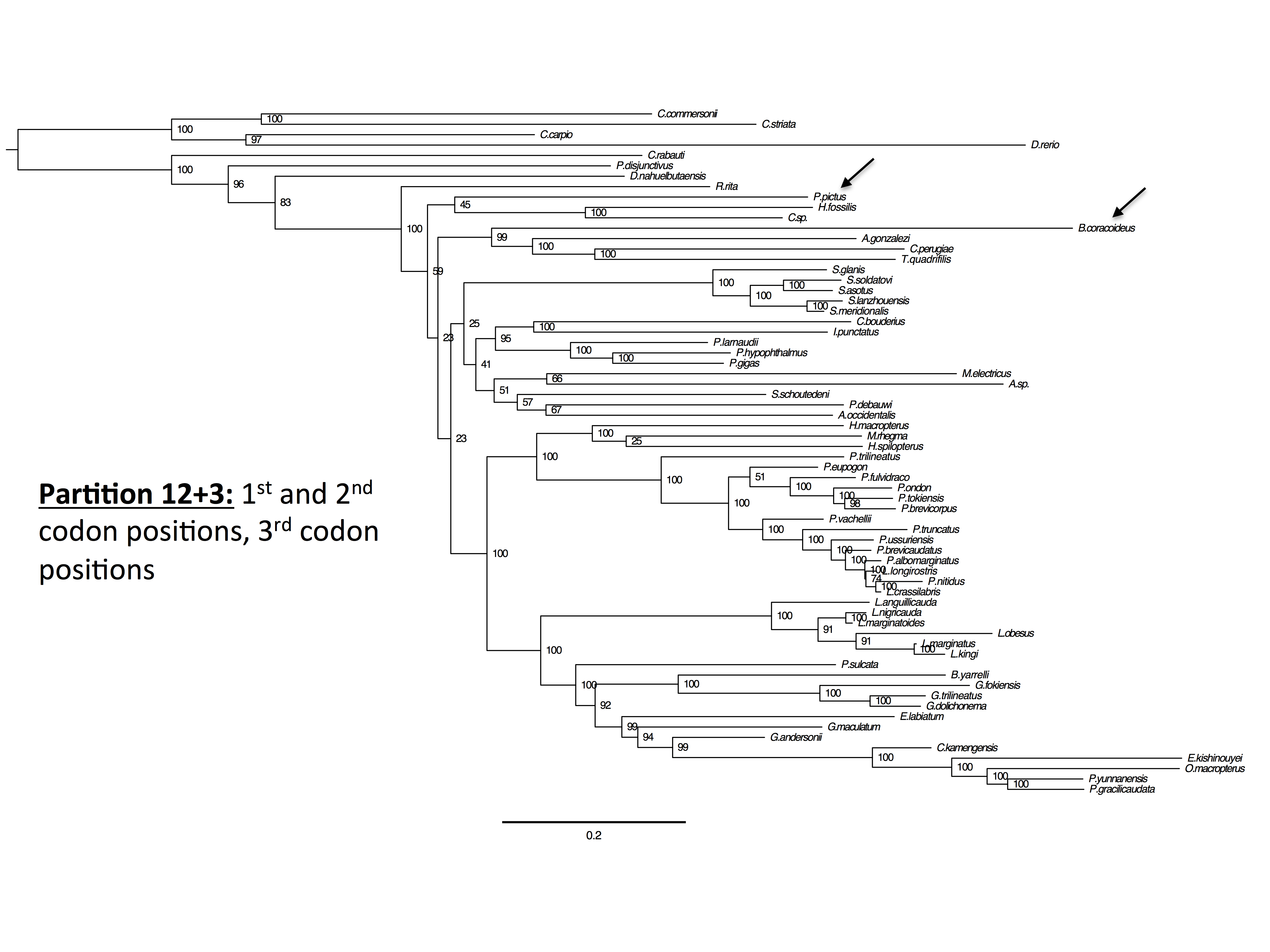

Supplement: S2 Fig — Branch lengths are proportional to the number of inferred substitutions. Numbers at nodes are bootstrap proportions based on 1,000 replicates. (TIFF) [file pone.0166988.s002.tiff]

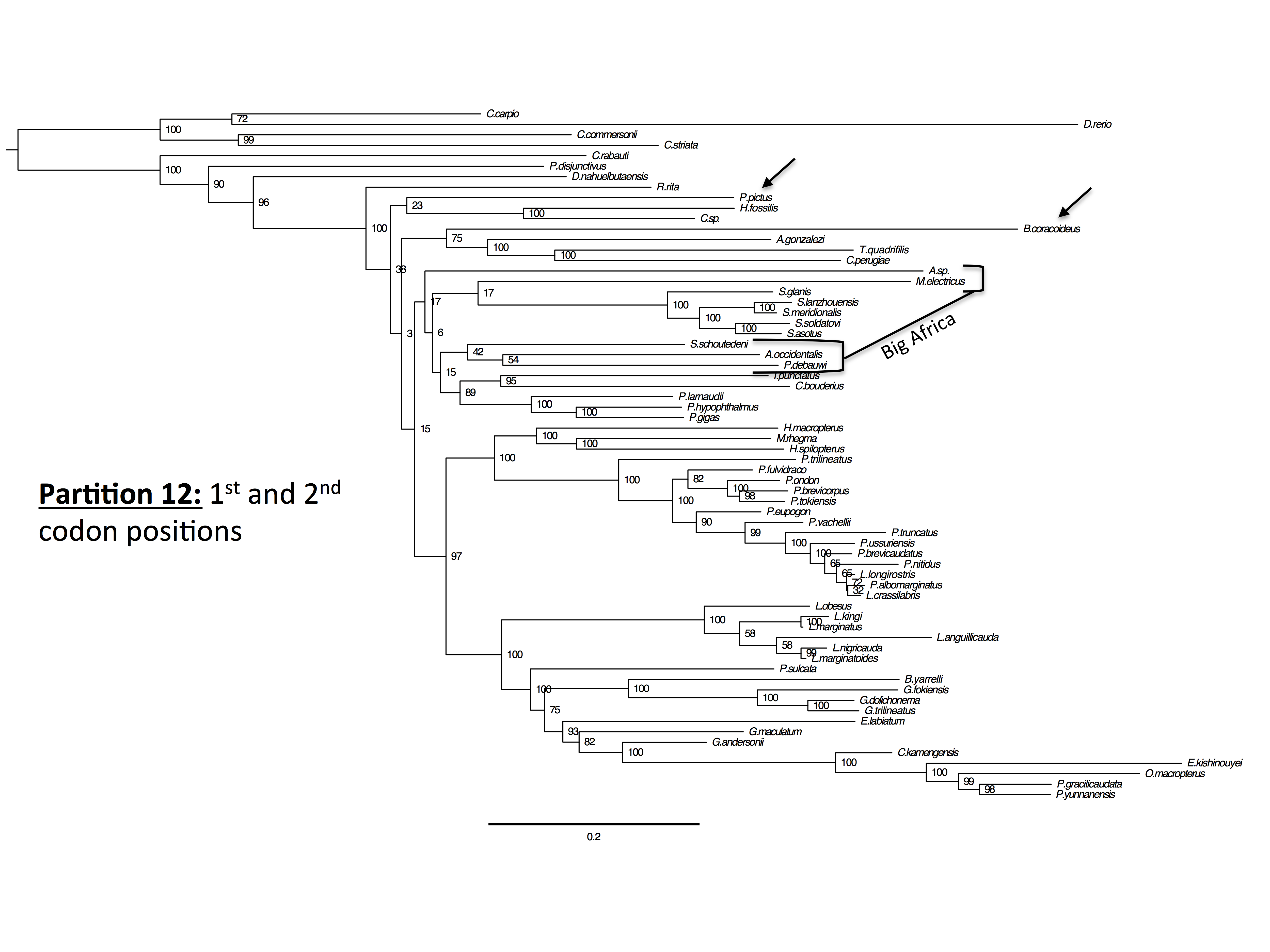

Supplement: S3 Fig — Branch lengths are proportional to the number of inferred substitutions. Numbers at nodes are bootstrap proportions based on 1,000 replicates. (TIFF) [file pone.0166988.s003.tiff]

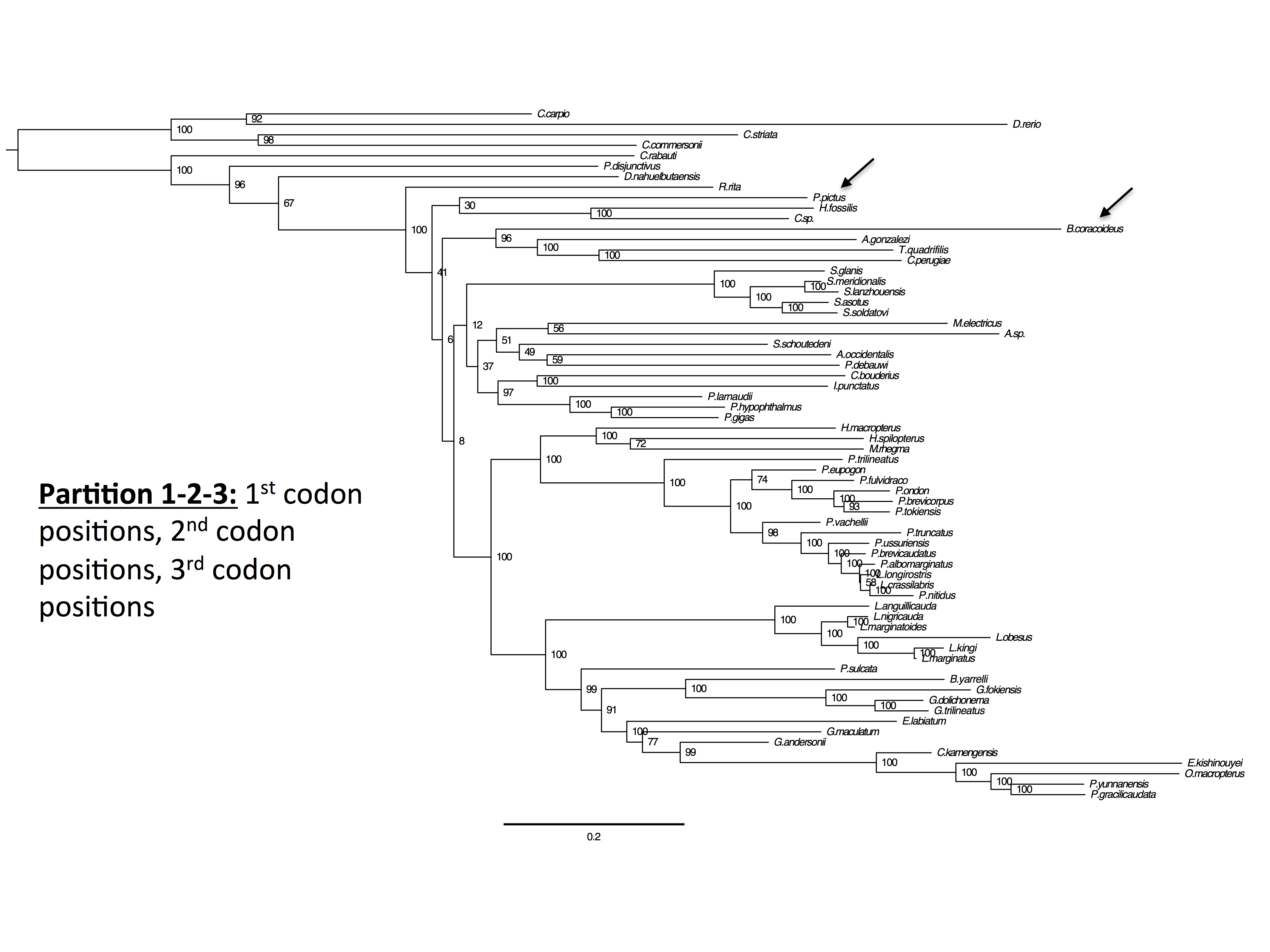

Supplement: S4 Fig — Branch lengths are proportional to the number of inferred substitutions. Numbers at nodes are bootstrap proportions based on 1,000 replicates. (TIFF) [file pone.0166988.s004.tiff]
